# Chelerythrine-Mediated Growth Inhibition and Resistance Mechanism in *Bacillus tropicus*

**DOI:** 10.3390/microorganisms13122731

**Published:** 2025-11-29

**Authors:** Jueyu Wang, Hongxia Wan, Wenqi Chai, Daizong Cui, Min Zhao

**Affiliations:** Key Laboratory for Enzyme and Enzyme-like Material Engineering of Heilongjiang, College of Life Science, Northeast Forestry University, Harbin 150040, China

**Keywords:** chelerythrine, *Bacillus tropicus*, drug resistance

## Abstract

Chelerythrine (CHE) is a naturally occurring benzophenanthridine alkaloid obtained from plants such as *Chelidonium majus* L. It has received notable attention in pharmacology and microbial control because of its broad-spectrum activity and marked anti-inflammatory, apoptosis-inducing, and antibacterial effects. In this study, *Bacillus tropicus*, which frequently presents in the soil environment, was selected as the target microorganism to systematically examine the dose-dependent inhibitory influence of CHE on its growth curve, biofilm development, and survival rate. Furthermore, by simulating an antibiotic pressure environment in vitro, the original strain was subjected to continuous subculturing (30 times), and a highly drug-resistant *B. tropicus* strain capable of stable growth under high concentrations of CHE (300 mg/L) was successfully acclimated. After that, transcriptomics analysis was employed to compare the genetic differences between the wild-type bacterium and drug-resistant bacterium to determine how bacterial cells are able to resist CHE. A total of 868 genes in the CHE-resistant bacterium were revealed to be more active, while 539 genes were less active. These results indicate that the CHE resistance characteristics of the strain may be related to the adjustment of its sugar metabolism pathway and the biofilm formation pathway. As a widely used biological control bacterial strain, the successful acclimation of the *B. tropicus* strain with resistance to CHE has made it possible to use the combined formulation of these two agents for the prevention and control of plant diseases.

## 1. Introduction

Agricultural practices currently involve the use of excessive artificial pesticides, which raises concerns about environmental pollution, harm to small animals other than pests, and effects on human health [1,2]. Therefore, it is imperative to identify better methods that are environmentally compatible and safer for humans, such as extracting useful natural substances from plants for use as pesticides [3,4,5]. Chelerythrine (CHE) is one such substance that is especially important in this regard. CHE is mainly derived from the *Macleaya cordata* (Willd.) R.Br. (*Papaveraceae*) plant and belongs to the category of benzophenanthridine alkaloids. CHE can fight against many bacteria, fungi, and pests that cause diseases in plants [6,7,8]. Thus, CHE is considered to be a potentially suitable botanical insecticide for crop protection.

However, the application of CHE in the soil is restricted because it is prone to degradation by environmental microorganisms, and the released products may be harmful to other organisms [9,10]. Moreover, some bacteria exhibit resistance to CHE [11,12,13]. For these reasons, CHE may be less effective in the field for long-term application. Therefore, understanding how some microorganisms adapt to CHE will better inform the study of its dynamics in the environment and enable the development of improved approaches for formulating pesticides containing this compound.

To date, most studies on microbial tolerance have focused on clinical pathogens and synthetic antibiotics, and little attention has been paid to the bacteria in the environment [14,15]. Soil is the most complex microbial community on earth, in which many microorganisms with unique metabolic abilities live in it. During the long-term evolution of these microorganisms, they have developed mechanisms to degrade and tolerate various plant secondary metabolites [16,17,18]. *Bacillus* species are particularly noteworthy in this regard because they adapt and grow fast, and many studies have reported their roles in promoting plant health and inhibiting soil-borne diseases [19,20]. Nevertheless, whether *Bacillus* strains can tolerate plant alkaloids such as CHE has not been systematically studied. This gap has hindered the full exploitation of these strains in sustainable agriculture and in biological control strategies based on natural products.

The antimicrobial properties of CHE present significant potential for its application as a biocontrol agent. However, a comprehensive evaluation must also consider its toxicity. As a kind of alkaloid compounds, CHE is known for their pharmacological activity and the potential to cause toxic effects on non-target organisms. However, its cytotoxicity represents a well-documented characteristic, and this toxic feature has also been explored in the context of its role as an anticancer agent. However, its cytotoxicity raises concerns regarding its environmental safety and potential ecological impact. Studies have shown that CHE induces oxidative stress and apoptosis across various types of cells. Shi et al. [21] demonstrated that exposure to CHE causes significant toxicity in zebrafish embryos by activating the cells’ oxidative stress and apoptosis pathways. This highlights the potential ecological risks brought about by the widespread use of CHE, emphasizing the necessity of conducting a comprehensive toxicological assessment before its wide application. Therefore, understanding the antibacterial effects, how to develop CHE resistance, and the balance of toxicity of CHE is crucial for the development of biopesticides based on CHE.

In this study, the inhibitory effect of CHE on the growth and metabolism of *B. tropicus*, which has strong environmental adaptability, was investigated, and the underlying drug resistance mechanism was also explored. A highly CHE-tolerant *B. tropicus* mutant was successfully domesticated through subculture on a medium supplemented with CHE at the MIC concentration for 30 generations. The physiological and biochemical responses to alkaloid stress were then studied, and the molecular basis of CHE tolerance was revealed through a transcriptome analysis. This study provides novel insights into the mechanism of microbial adaptation and opens up new avenues for the application of these strains as biological control agents, biological mediators, or synergists in the development of next-generation plant protection products. Finally, the combination of microbial innovation and natural product utilization employed in this study would help to promote the sustainable agricultural practices.

## 2. Materials and Methods

### 2.1. Chemicals and Materials

*B. tropicus* (designated as strain BT) was previously isolated from a soil sample collected in a forest area near a farmland, Heilongjiang Province, China. The strain is stored in the Strain Preservation Center of Northeast Forestry University with the number of ZL6.

CHE with a purity of 98% was obtained from Nanjing Daosifu Technology Co., Ltd. (Nanjing, China), and 4′,6-diamidine-2-phenylindole (DAPI) was purchased from Solarbio Science & Technology Co., Ltd. (Beijing, China). All other chemicals and reagents were of analytical grade.

### 2.2. Minimum Inhibitory Concentrations (MICs) of Wild-Type B. tropicus (BT)

The MIC values were determined using the broth microdilution method following the guidelines of the Clinical and Laboratory Standards Institute (CLSI) [22]. Briefly, the strain *B. tropicus* was first inoculated into the LB medium and cultured until it reached its logarithmic growth phase. After centrifugation at 4 °C at 10,000 r/min for 5 min, the bacterial cells were collected and resuspended using sterilized LB medium to make sure that the concentration of the bacterial cells in the culturing system was 5 × 10^7^ CFU/mL. After that, 100 μL of this suspension culture was added to each well of a 96-well plate. CHE (with the final concentrations of CHE at 1.25, 2.5, 5, 10, 20, 40, 80, 160 mg/L) was then added to the bacterial suspensions. The OD_600_ in each well was measured after 24 h of incubation at 37 °C under shaking conditions. After incubation, the MIC was defined as the lowest concentration of CHE that completely inhibited visible bacterial growth, which was assessed spectrophotometrically by measuring the optical density at 600 nm (OD_600_).

### 2.3. Domestication of Drug-Resistant B. tropicus (CheRBT)

Wild-type *B. tropicus* (BT) was subcultured with half of the MIC of CHE as the initial concentration, and the MIC value was determined every five generations. If the MIC of CHE for *B. tropicalis* changed or increased, the concentration of CHE was readjusted.

### 2.4. Differential Growth and Metabolism of BT and CheRBT

#### 2.4.1. Growth Curve Determination

Cultures of wild-type and CHE-resistant B. tropicalis were diluted to an initial OD_600_ of 0.1. The final concentration of CHE in the bacterial suspension was prepared to 25 mg/L, with a control group also set up for comparison. All the cultures were incubated at 37 °C, and OD_600_ values were measured at 0, 2, 4, 6, 8, 10, 12, and 24 h, respectively, to monitor the bacterial growth rate.

#### 2.4.2. Scanning Electron Microscopy (SEM)

The log-phase BT and CheRBT cultures (OD_600_ = 0.1) were treated with CHE at the MIC for 4 h. The cells were collected, washed, fixed with glutaraldehyde, dehydrated in ethanol, freeze-dried, sputter-coated with gold, and observed using SEM.

#### 2.4.3. Biofilm Formation

The OD_600_ values of the log-phase cultures of BT and CheRBT were adjusted to 0.1 using fresh medium. A 100 μL portion of each suspension was transferred into the wells of a 96-well plate and incubated at 37 °C for 24 h to permit biofilm development. After incubation, the planktonic cells were discarded, and the attached biofilms were rinsed three times with 150 μL of PBS, followed by air-drying. The biofilms were subsequently fixed with 150 μL of methanol for 5 min and stained with 100 μL of crystal violet for 20 min. Excess dye was removed by washing with sterile water until the effluent became colorless. The crystal violet associated with the biofilm was then dissolved in 33% glacial acetic acid for 20 min, and the OD_570_ of the final solution was recorded.

#### 2.4.4. Glucose Content in the BT and CheRBT Cultures

In order to assess the effects of CHE on glucose utilization, cultures of BT and CheRBT were inoculated separately into LB media supplemented with 1 g/L glucose, and OD_600_ was adjusted to 0.1. CHE was subsequently added to the media to achieve a final concentration of 25 mg/L. The cultures were then incubated at 37 °C, and samples were collected at 0, 2, 4, 6, and 8 h, respectively. After centrifugation at 7500 r/min for 10 min, the glucose concentration in the resulting supernatants was quantified using the 3,5-dinitrosalicylic acid (DNS) method.

#### 2.4.5. BT and CheRBT DNA Content

In order to observe the cellular DNA content, BT and CheRBT were diluted to reach an OD_600_ of 0.1. CHE was added to reach a final concentration of 25 mg/L, and the mixture was incubated at 37 °C for 4 h. Subsequently, the cells were stained with DAPI through incubation in the dark for 10 min to visualize the DNA. A 5 µL aliquot of the stained suspension was placed on a microscope slide, which was observed using confocal laser scanning microscopy (CLSM) by ZEISS LSM 510 META (Oberkochen, Germany).

### 2.5. Transcriptome Analysis

#### 2.5.1. RNA Preparation, Library Construction, and Sequencing

Total RNA was extracted from BT and CheRBT cells according to the instructions of the RNA extraction kit, and RNA quality was assessed using Nanodrop by Guangdong Denley Technology Co., Ltd. (Guangzhou, China) and agarose gel electrophoresis. After confirming RNA integrity, a cDNA library was constructed from the RNA sample. Ribosomal RNA (rRNA) was first removed using probe hybridization, which was followed by fragmentation of the remaining RNA. The fragmented RNA was then reverse-transcribed into double-stranded cDNA using random primers, and the resulting cDNA was purified, end-repaired, A-tailed, ligated with sequencing adapters, and size-selected. The library was ultimately enriched through PCR amplification. The library concentration was measured using a Qubit 2.0, and the effective concentration was quantified using Q-PCR to ensure quality. After validation, high-throughput sequencing was performed on the HiSeq 2500 platform.

#### 2.5.2. Read Mapping, Annotation, and Analysis

The raw data were processed to remove the adapter sequences and low-quality reads, generating high-quality clean data. Using Rockhopper 2, the clean reads were aligned to the designated reference genome, generating positional information on the genome or genes along with sequence-specific features unique to the sequenced samples. In order to determine the gene expression levels, the reads from each sample were aligned to the predicted genes using BLAT, and the expression levels were quantified based on these alignment results. The abundance of each gene was represented by its FPKM value. Gene Ontology (GO) enrichment analysis was conducted; the number of the differentially expressed genes (DEGs) associated with each GO term was counted, and the significance of their enrichment was calculated using the hypergeometric distribution algorithm. Pathway analysis of the differentially expressed protein-coding genes was performed next using the KEGG database (in conjunction with the KEGG annotation results). The significance of DEG enrichment in each pathway was also calculated using the hypergeometric distribution test [23,24].

#### 2.5.3. Defining and Analyzing the Differentially Expressed Genes

In order to identify the differentially expressed protein-coding genes, differential expression analysis was performed using the DESeq2 R (v 3.2.0). Initially, the raw gene count data for each sample were normalized to correct for library size and other systematic biases, and expression levels were evaluated using the BaseMean value. After that, DESeq2 applied a negative binomial distribution model to estimate fold change (FC) and assess the statistical significance of differential gene expression. Genes were ultimately defined as significantly differentially expressed when they satisfied the strict thresholds of an absolute fold change greater than 2 (|FC| > 2) and an adjusted *p* value (FDR) below 0.05.

### 2.6. Statistical Analysis

Data are presented as mean ± standard deviation (SD) from at least three independent experiments. Comparisons between two groups were performed using Student’s *t*-test, while comparisons among multiple groups were conducted using one-way analysis of variance (ANOVA) followed by Tukey’s post hoc test. A *p*-value of less than 0.05 (*p* < 0.05) was considered statistically significant.

## 3. Results and Discussion

### 3.1. MICs of the Wild-Type and Chelerythrine-Resistant B. tropicus Strains

**Figure 1:**
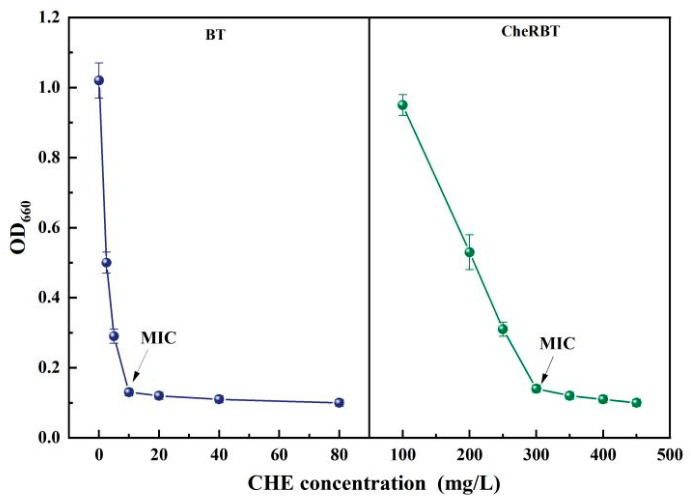
CHE MIC of the drug-resistant and wild-type strains of *B. tropicus*.

The MIC of CHE against the wild-type B. tropicus (BT) strain was 10 mg/L. In contrast, the CHE-resistant strain (CheRBT) exhibited an MIC of 300 mg/L, representing a 30-fold increase in resistance compared to the wild-type (Figure 1). This substantial elevation in MIC confirms the successful induction of a stable, high-level resistant phenotype in the laboratory, indicating that *B. tropicus* can rapidly adapt to the selective pressure imposed by CHE. It has been reported that some bacterial strain exhibited highly resistance effects on the biological alkaloids extracted from plants. For example, berberine stimulates biofilm production in *Escherichia coli* by upregulating the *csgD* gene, establishing a protective barrier that enhances the community’s drug tolerance [25]. In our study, the MIC of CheRBT was 300 mg/L, which has already far exceeded its resistance to other biological control pesticides. These results fully demonstrate that CheRBT adapt well with the environment containing high concentration of CHE and has a big potential to be formulated together with CHE.

The antibacterial properties of CHE are not limited to the *B. tropicus* strain alone. This benzo[c]phenanthridine alkaloid has been widely reported to have broad-spectrum antibacterial activity against various microorganisms, especially on Gram-positive bacteria (such as *Staphylococcus aureus* and *Bacillus subtilis*), and it also has inhibiting effects on fungi (such as *Candida albicans*) [9]. CHE can disrupt the microbial cell membrane and interfere with key metabolic pathways of the microbial cells, which lays the foundation for its effectiveness as an antibacterial agent. Therefore, understanding how bacteria like *B. tropicus* develop resistance to this antibacterial agents is crucial for evaluating its potential application value and managing the antimicrobial resistance.

### 3.2. Growth and Metabolism Changes in BT and CheRBT

#### 3.2.1. Growth Curve Changes in CheRBT with CHE Addition

**Figure 2:**
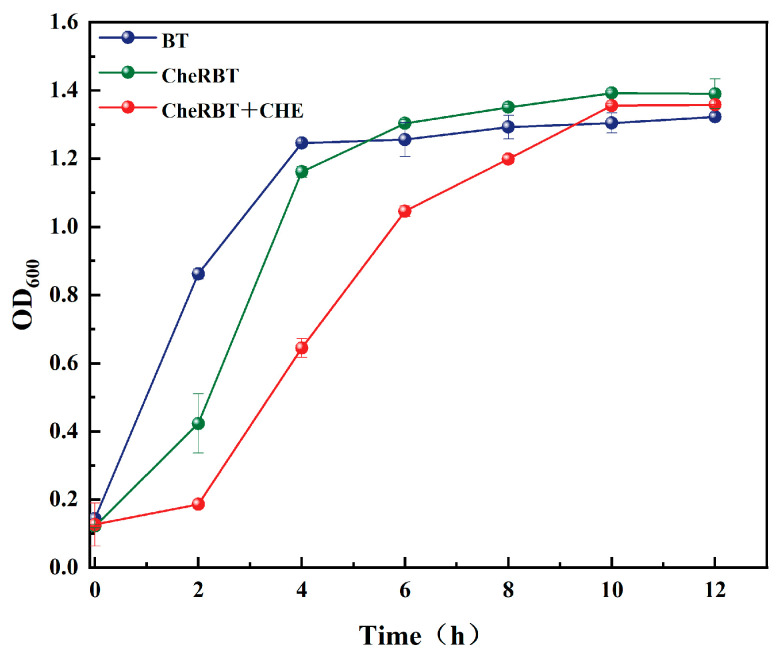
Growth curves of the drug-resistant and wild-type strains of *B. tropicus*.

The growth kinetics of *B. tropicus* (BT) and the CHE-resistant strain (CheRBT) were compared, as depicted in Figure 2. The results indicate that BT exhibited a shorter lag phase and entered the exponential growth phase earlier than CheRBT. Despite this delayed onset of exponential growth, CheRBT ultimately reached a stationary phase with a maximum optical density comparable to that of the wild-type strain. This suggests that the presence of CHE does not inhibit the maximum growth yield of CheRBT, but rather delays the initiation of its exponential proliferation.

#### 3.2.2. Morphological Characteristics of the Two Strains

**Figure 3:**
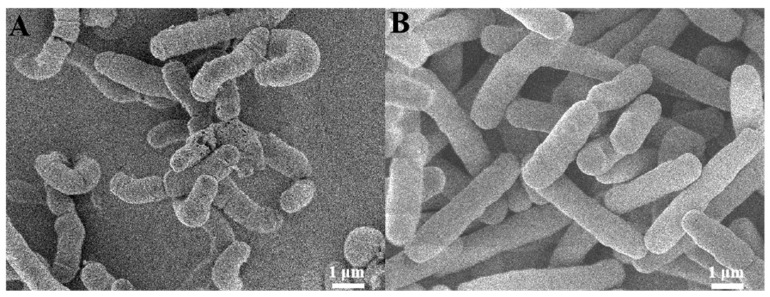
SEM images of (**A**) BT and (**B**) CheRBT.

Morphological analysis revealed significant differences between BT and CheRBT. As shown in Figure 3, compared with the wild-type cells, the CheRBT cells were notably elongated, with rounded ends and a smoother surface. At the same time, biofilm quantification (Figure S1 and Table S1) revealed that CheRBT formed about 14.1% more biofilm than the wild-type strain. These findings suggested two potential, non-exclusive mechanisms for CHE resistance in CheRBT: morphological adaptation and enhanced biofilm production. Alterations in cell morphology can be a stress response to toxic compounds, whereas increased biofilm formation is a common strategy that enhances bacterial resistance to antimicrobials [26]. The concurrent observation of both phenotypes in CheRBT indicates a multifaceted adaptive strategy in this bacterium to withstand CHE.

#### 3.2.3. Glucose Content in the BT and CheRBT Culturing Systems

**Figure 4:**
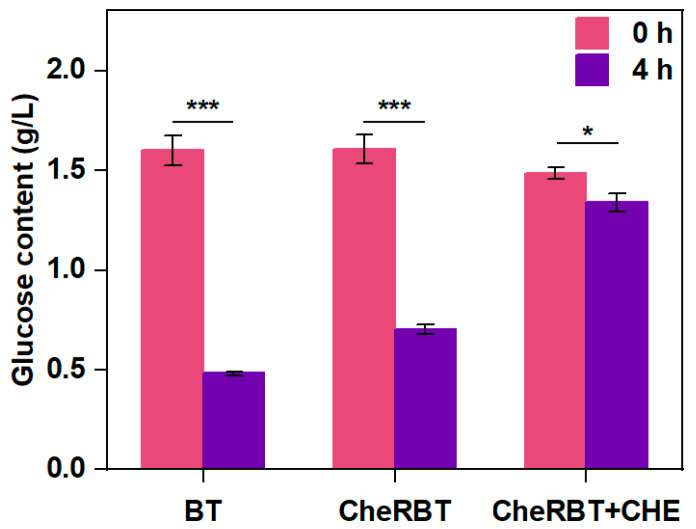
Glucose content in the media for BT and CheRBT culturing (*** *p* ≤ 0.001, * *p* ≤ 0.05).

Glucose is one of the common preferred carbon sources for bacteria [27]. As shown in Figure 4, the two bacteria consumed glucose in different ways. After 4 h of culturing, BT consumed 70% of the available glucose, which was much faster than the rate CheRBT consumed (55.6% glucose consumed with the same period). It was interesting to see that CHE further prevented CheRBT from consuming glucose, with a consumed amount of only 10.8%. These findings indicated that at the beginning of the incubation (0–4 h), BT consumed glucose faster than CheRBT, and CHE further prevented CheRBT from consuming a similar amount of glucose as done by BT. This finding also explains the growth situation observed: the rapid growth stage of CheRBT with CHE was delayed, possibly due to the delayed glucose consumption. In brief, at the beginning of growth, BT consumed glucose faster than CheRBT, and CHE slowed down the rate at which CheRBT consumed glucose.

#### 3.2.4. DNA Content of BT and CheRBT

**Figure 5:**
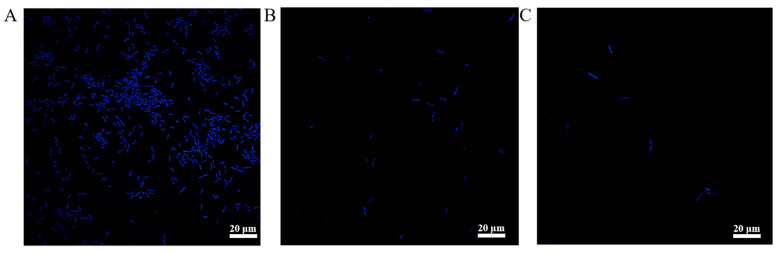
Glucose content of (**A**) BT, (**B**) CheRBT, and (**C**) CheRBT with CHE addition.

DAPI combines with DNA, resulting in a unique blue fluorescence [28]. This characteristic was utilized to assess the integrity and permeability of the bacterial cells. As shown in Figure 5, the intensity of the fluorescence emitted by different strains varied greatly. The fluorescence emitted by BT (Figure 5A) was much stronger than that emitted by CheRBT (Figure 5B). Moreover, even after being treated with CHE, the fluorescence of CheRBT (Figure 5C) did not change much compared with that of the untreated sample. The DAPI signal of CheRBT was weakened, which indicated that something was blocking the dye from entering the cell. We hypothesized that this obstacle was related to the phenotypic characteristics of CheRBT previously observed: enhanced biofilm formation and/or spore formation ability. Thicker biofilms or spores can act as a shield, physically blocking DAPI, not allowing it to make contact with DNA inside cells, resulting in weaker fluorescence signals.

### 3.3. Transcriptome Analysis

#### 3.3.1. Identification of Differentially Expressed Genes (DEGs)

**Figure 6:**
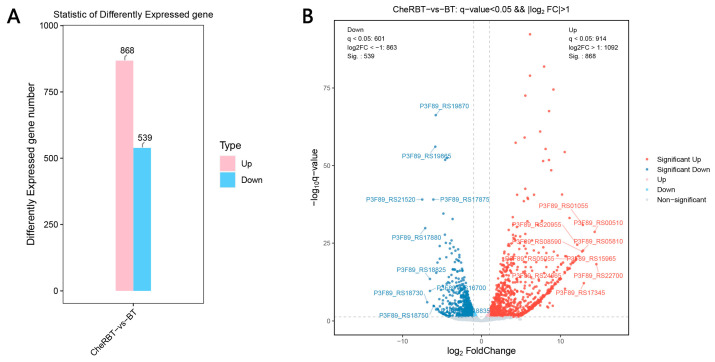
Gene expression differences between the comparison groups: (**A**) bar chart and (**B**) volcano plot.

Transcriptome analysis provides a comprehensive understanding of gene expression through the analysis of all RNA transcripts of the cells under specific conditions, including mRNAs, rRNAs, tRNAs, and siRNAs [29]. This method can not only measure the transcriptional response of cells to the environmental stimuli but also helps decipher the molecular mechanism that causes these changes [30]. In this study, a comparative transcriptome analysis was conducted to identify DEGs. As shown in Figure 6A, after comparing the CheRBT group with the BT group, 868 upregulated and 539 downregulated DEGs were identified. To illustrate the extent and statistical relevance of these transcriptional alterations, volcano plots were constructed for both comparisons (Figure 6B). In these plots, each dot corresponds to a gene, with its location reflecting the fold change (log2 scale) and statistical significance (−log10 *p* value). Genes are color-marked, with red indicating upregulated genes, blue indicating downregulated genes, and gray indicating changes without statistical significance. This representation clearly reveals the distinct transcriptional profiles among the sample groups.

#### 3.3.2. GO Enrichment Analysis

**Figure 7:**
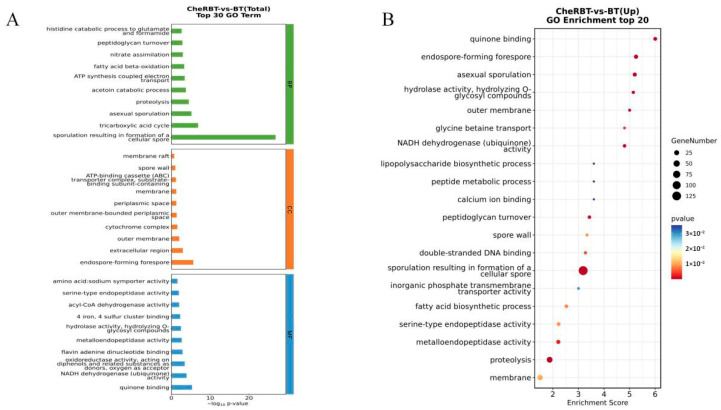
Differentially expressed gene set GO classification chart: (**A**) bar chart and (**B**) bubble chart.

A Gene Ontology (GO) enrichment analysis was performed on the DEGs identified in the CheRBT and BT groups (Figure 7). In total, 1024 DEGs were mapped to 359 GO terms (173 biological process, 30 cellular component, and 156 molecular function terms). The key enriched terms included sporulation (152 DEGs), proteolysis (58 DEGs), and a downregulated tricarboxylic acid cycle (18 DEGs) in the biological process category. In the Cellular Component category, the significant terms were the extracellular region (48 DEGs) and forespore (14 DEGs, all upregulated). The Molecular Function category had significant enrichment in metal ion binding (127 DEGs), 4 iron, 4 sulfur cluster binding (27 DEGs), and metalloendopeptidase activity (20 DEGs), along with several upregulated hydrolase and dehydrogenase activities.

#### 3.3.3. KEGG Pathway Analysis

**Figure 8:**
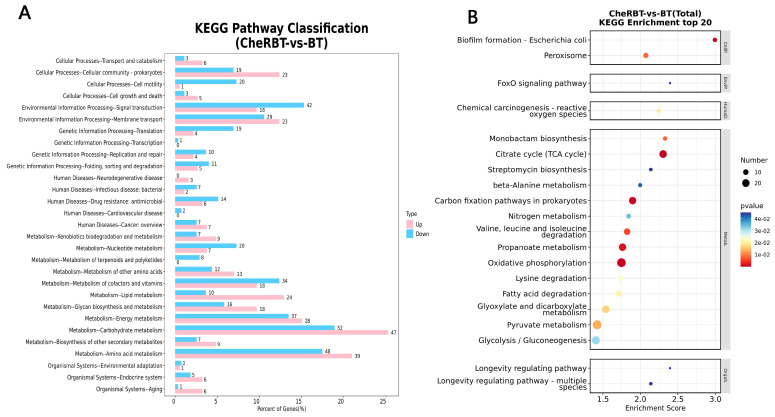
Histogram of the KEGG pathway classification of the differentially expressed genes. (**A**) Bar chart. (**B**) Bubble chart.

KEGG pathway annotation of the DEGs between the CheRBT and BT groups (Figure 8) classified the DEGs into six categories: metabolism (463 DEGs), environmental information processing (112 DEGs), cellular processing (80 DEGs), genetic information processing (54 DEGs), human diseases (48 DEGs), and organismal systems (21 DEGs). In the metabolism category, the DEGs were involved mainly in carbohydrate (99 DEGs), amino acid (87 DEGs), and energy (65 DEGs) metabolism. In the context of environmental information processing, signal transduction (60 DEGs) and membrane transport (52 DEGs) were prominent. Cellular processes were dominated by the terms “cellular community” (42 DEGs) and “cell motility” (21 DEGs). Genetic information processing primarily included translation (23 DEGs), folding, sorting, and degradation (16 DEGs), and replication and repair (14 DEGs). Organismal system DEGs were involved in the endocrine system (11 DEGs), aging (7 DEGs), and environmental adaptation (3 DEGs).

### 3.4. Drug Resistance Mechanism Analysis

#### 3.4.1. Glycolysis and Gluconeogenesis

KEGG pathway enrichment analysis revealed that the DEGs in the CheRBT vs. BT comparison group were significantly enriched in the glycolysis and gluconeogenesis pathways. The specific DEGs are listed in Table S2. CheRBT downregulated the glycolysis pathway while upregulated the gluconeogenesis pathway. Specifically, the downregulation of the PTS glucose transporter in CheRBT impeded glucose uptake. Concurrently, the inhibition of key glycolytic enzymes (such as glucokinase, glucose-6-phosphate isomerase, 6-phosphofructokinase, and pyruvate kinase) reportedly blocks the glycolytic process [31]. The upregulation of AMP-binding proteins allows the bacteria to sense a low-energy state, thereby activating gluconeogenesis and fermentation pathways [32]. The upregulation of L-lactate dehydrogenase, bifunctional acetaldehyde-CoA, and zinc-dependent alcohol dehydrogenase facilitates the conversion of pyruvate into products such as lactate and ethanol. The key enzymes in the gluconeogenesis pathway, such as class II fructose-bisphosphate aldolase, were upregulated, thus enhancing this pathway and enabling bacteria to synthesize glucose from the non-carbohydrate substrates. The glucose synthesized using gluconeogenesis may be further used to produce glycogen, as evidenced by the upregulated expression of glycogen synthase. Jackson [33] demonstrated that glycogen biosynthesis and its subsequent turnover are essential for biofilm formation, which is consistent with the biofilm formation pathway analysis results of this study. Owing to the downregulation of the pyruvate dehydrogenase complex, the entry of metabolites into the TCA cycle was inhibited in CheRBT, leading to a downregulation trend in the TCA cycle, which is consistent with the results of the citrate cycle analysis presented ahead. The enhancement of the gluconeogenesis pathway may alter the bacterial energy distribution to help improve tolerance to CHE, or the intermediates of this pathway may participate in antioxidant defense, thus mitigating the CHE-induced oxidative stress.

#### 3.4.2. Biofilm Formation Analysis

KEGG pathway enrichment analysis revealed that, compared with those in the BT group, the DEGs in the CheRBT group were significantly enriched in pathways related to biofilm formation and tended to be upregulated overall (Table S3). These changes in gene expression suggested that CheRBT promotes biofilm formation by regulating glycogen synthesis and degradation, as well as through quorum-sensing mechanisms, to coordinate the collective bacterial behavior [34]. Specifically, activities of the genes related to glycogen metabolism may provide the necessary energy and carbon sources for the growth and maintenance of biofilms so that the bacteria can continue to grow and maintain their structural stability in poor environments. Balestrino [35] noted that the lack of the luxS gene leads to an obvious thinning of biofilms, as this gene can coordinate the collective action of bacteria by regulating the synthesis of the quorum-sensing signal molecule AI-2. The quorum-sensing-related genes observed in this study became active, possibly through strengthening of the AI-2 signaling pathway, thus promoting the formation and stability of biofilms. In addition, activities of the genes related to glucose absorption decreased, indicating that the bacteria changed their energy strategy when glucose was insufficient and reduced their dependence on the traditional glucose metabolism pathways, such as glycolysis and the TCA cycle, turning to glycogen metabolism [36]. This change in the metabolic mode enabled the bacteria to form a stable biofilm structure even in undernourished or complex environments, thereby greatly enhancing their adaptation to the environment, viability, and drug resistance.

#### 3.4.3. TCA Cycle

Compared with those in the BT group, DEGs in the CheRBT group were significantly enriched in the TCA cycle pathway and tended to be downregulated overall (Figure S2). Among these genes, only the citrate synthase gene (*mmgD*) was upregulated, whereas multiple key enzyme-encoding genes, including citrate synthase (*citZ*), NADP-dependent isocitrate dehydrogenase (*icd*), malate dehydrogenase (*mdh*), succinate dehydrogenase subunits (*sdhA*, *sdhB*, *sdhC*), pyruvate dehydrogenase complex subunits (*pdhA*, *pdhB*, *pdhC*, *lpdA*), succinate-CoA ligase subunits (*sucC*, *sucD*), aconitate hydratase (*acnA*), the 2-oxoglutarate dehydrogenase complex subunits (*odhA*, *odhB*), and class I fumarate hydratase (*fumA*), were significantly downregulated. Although the upregulation of mmgD could partially promote the initiation of the TCA cycle, the downregulation of most of the key enzymes indicated that the overall function of the TCA cycle in CheRBT was markedly suppressed. Sylvain [37] reported that in Pseudomonas aeruginosa, metabolites derived from the lower section of the TCA cycle (including fumarate and succinate) and from the lower section of glycolysis (including pyruvate and acetate) increased susceptibility to tobramycin, while metabolites from the upper TCA cycle (such as citrate and glyoxylate) showed minimal influence. Based on these observations, we proposed that CheRBT may acquire tolerance to CHE by suppressing metabolic pathways in the lower TCA cycle, thereby decreasing its sensitivity to CHE. In addition, previous studies have indicated that bacteria can enter a metabolically inactive state and generate persister cells by reducing TCA cycle activity under environmental stress. CheRBT may have employed a comparable mechanism to develop tolerance to CHE.

In our study, the strain CheRBT may cope with the stress of the antibiotic CHE by inhibiting its activity of core metabolic pathways, such as the TCA cycle. This significant downregulation of metabolic activity is considered a key strategy for bacteria to enter a state of metabolic dormancy and form persister cells [1]. In this dormant state, the growth and division of cells are very slow, thereby avoiding the lethal effects of CHE that primarily target active cellular processes (e.g., the cell wall synthesis process and the DNA replication process). The persister cells of CheRBT have low ability for ATP generation, which affects the efficiency of antimicrobial chemicals in being transported into the cell through an energy-consuming transport process. Simultaneously, the stress of this metabolic change can activate broader responses like the stringent response, pushing the cells to go into a deeper dormant state.

Besides the state of metabolic dormancy, alterations in cellular metabolism can also modulate antimicrobial susceptibility through changes in the cell membrane. First, efflux pumps are critical weapons for bacteria to extrude antibiotics and develop multidrug resistance, and their function is an energy-dependent active transport process. The changes in glycolysis and TCA cycle activity observed in this study directly impact the ATP supply. If the CheRBT strain reallocates its limited energy preferentially to power the efflux pump systems instead of growth and proliferation, it could effectively increase the excretion rate of substrates such as CHE. Second, shifts in metabolic patterns are also linked to the modification of the physicochemical properties of the cell membrane. To counteract nutrient limitation or antibiotic stress, bacteria alter their phospholipid composition by increasing hydrophobic and branched-chain fatty acids [2]. This adaptation enhances membrane integrity, reduces permeability, and may restrict CHE entry. Together with structural biofilm changes, these membrane modifications form a potent physicochemical shield, which leads to the strain has a higher resistance to CHE.

## 4. Conclusions

A CHE-resistant Bacillus strain has been successfully acclimated through serial passaging. Compared with the wild-type strain, CheRBT resulted in a 30-fold increase in the MIC of CHE. Growth curve analysis revealed that while CheRBT has a lower growth rate during the logarithmic growth phase, it reaches the stationary phase and peak biomass at a similar time as the wild type. Morphological analysis revealed that the tolerance of CheRBT may be related to changes in cell structure and increased biofilm formation ability. Transcriptome analysis revealed the molecular basis of this tolerance, which involves upregulation of the genes related to spore formation and biofilm formation, as well as regulating the efficiency of key metabolic pathways (such as citrate cycle, glycolysis, and gluconeogenesis pathways). Therefore, it was speculated that the tolerance of CheRBT to CHE is established through multiple strategies, including morphological changes, metabolic changes, biofilm formation ability changes, and so on.

## Data Availability

The original contributions presented in this study are included in the article and Supplementary Materials. Further inquiries can be directed to the corresponding authors.

## Supplementary Materials

The following supporting information can be downloaded at: https://www.mdpi.com/article/10.3390/microorganisms13122731/s1, Figure S1: Figure 3 and Figure 4 Biofilm Formation rate; Figure S2: Citric acid cycle signaling pathway; Table S1: UV spectrophotometer values for biofilm staining; Table S2: Genes with Significant Difference in Glycolysis and gluconeogenesis; Table S3: Genes with Significant Difference in Biofilm formation.
