# Supplementary material for: Chelerythrine-Mediated Growth Inhibition and Resistance Mechanism in Bacillus tropicus
Source: Microorganisms. 2025 Nov 29;13(12):2731. doi: 10.3390/microorganisms13122731 (PMC12735741; doi:10.3390/microorganisms13122731)
Supplement: Supplementary file 1 [file microorganisms-13-02731-s001.zip › microorganisms-3976708-supplementary.pdf]

Chelerythrine-Mediated Growth Inhibition and Resistance Mechanism in *Bacillus tropicus*

Jueyu Wang, Hongxia Wan, Wenqi Chai, Daizong Cui * and Min Zhao *

Key Laboratory for Enzyme and Enzyme-like Material Engineering of Heilongjiang, College of Life Science, Northeast Forestry University, Harbin 150040, China

* Correspondence: siyu19831114@163.com (D.C.); 82191513@163.com (M.Z.)

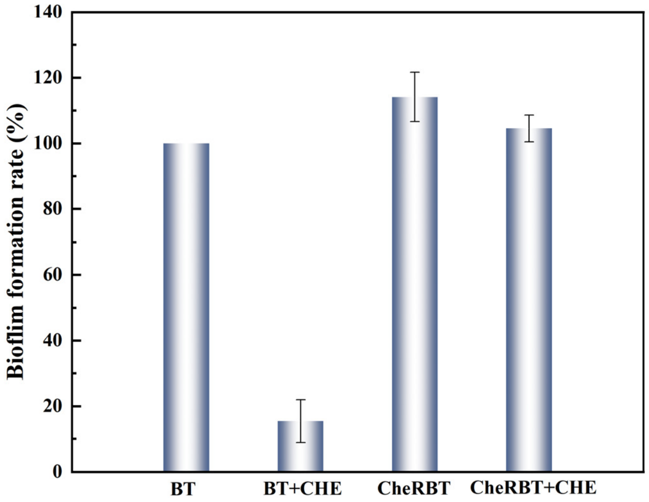

Figure S1: Figures 3 and 4 Biofilm Formation rate

Table S1 UV spectrophotometer values for biofilm staining

	BT	BT+CHE	CheRBT	CheRBT+CHE
OD570nm	3.047±0.038	0.471±0.066	3.476±0.076	3.184±0.004

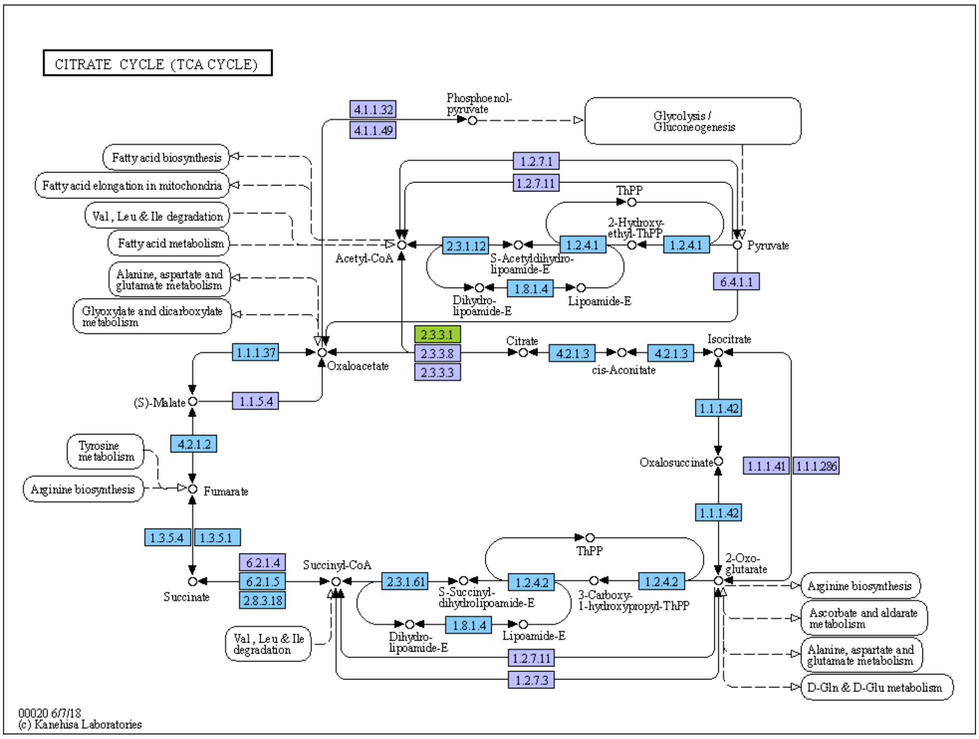

Figure S2: Citric acid cycle signaling pathway

Table S2 Genes with Significant Difference in Glycolysis and gluconeogenesis

Gene id	Gene name	CheRBT - Vs - BT log2(FC)	P value （P＜0.05）
P3F89_RS00780		1.137059437	1.56104e^{-05}
P3F89_RS02415		3.115252101	9.88982e^{-10}
P3F89_RS04055	*acsA*	2.178457577	1.2045e^{-09}
P3F89_RS04150	*mbcS*	2.945347227	2.47538e^{-13}
P3F89_RS05495	*adhE*	2.231195155	3.71438e^{-08}
P3F89_RS10000	*dhaS*	2.434746388	5.97514e^{-11}
P3F89_RS11085		4.543073718	0.019290572
P3F89_RS12200		1.790312383	0.014402192
P3F89_RS13545		3.98320777	2.70035e^{-30}
P3F89_RS14920		2.784179199	2.61387e^{-16}
P3F89_RS17690		2.561413714	1.5807e^{-07}
P3F89_RS00860		-1.358683363	4.09825e^{-05}
P3F89_RS02775		-1.562599041	1.15009e^{-05}
P3F89_RS04400	*pfkA*	-1.222931671	5.68364e^{-05}
P3F89_RS04405	*pyk*	-1.675638283	5.64815e^{-07}
P3F89_RS06025	*glcK*	-2.204728724	1.25663e^{-07}
P3F89_RS07120	*ptsG*	-1.146674875	0.001226016
P3F89_RS07540	*pdhA*	-1.227414133	0.000226455
P3F89_RS07545	*pdhB*	-1.578940359	4.8544e^{-06}
P3F89_RS07550	*pdhC*	-1.319090594	9.73573e^{-05}

P3F89_RS13845		-2.668298403	$1.44072e^{-12}$
P3F89_RS13850	*lpdA*	-2.906145557	$1.71919e^{-15}$

Table S3 Genes with Significant Difference in Biofilm formation

Gene id	Gene name	CheRBT - Vs - BT log2(FC)	P value （P＜0.05）
P3F89_RS02950	*glgC*	3.43974517	$3.44917e^{-27}$
P3F89_RS02955	*glgD*	4.049553524	$2.80226e^{-29}$
P3F89_RS02960	*glgA*	2.512200254	$4.32833e^{-14}$
P3F89_RS02965	*glgP*	2.133785645	$1.86023e^{-08}$
P3F89_RS03330	*luxS*	2.80170174	$1.39209e^{-08}$
P3F89_RS12780		10.05874953	$1.89788e^{-15}$
P3F89_RS00860		-1.358683363	$4.09825e^{-05}$
